# Design of simulation-based medical education and advantages and disadvantages of in situ simulation versus off-site simulation

**DOI:** 10.1186/s12909-016-0838-3

**Published:** 2017-01-21

**Authors:** Jette Led Sørensen, Doris Østergaard, Vicki LeBlanc, Bent Ottesen, Lars Konge, Peter Dieckmann, Cees Van der Vleuten

**Affiliations:** 1 Juliane Marie Centre for Children, Women and Reproduction, Rigshospitalet, University of Copenhagen, 2100 Copenhagen, Denmark; 2 Copenhagen Academy for Medical Education and Simulation, Herlev Hospital, Capital Region of Denmark and University of Copenhagen, 2730 Herlev, Denmark; 3 Department of Innovation in Medical Education, University of Ottawa, Ottawa, Canada; 4 University of Ottawa Skills and Simulation Centre, The Ottawa Hospital, & University of Ottawa, K1Y 4E9 Ottawa, Canada; 5 Copenhagen Academy for Medical Education and Simulation, Rigshospitalet, Capital Region of Denmark and University of Copenhagen, 2100 Copenhagen, Denmark; 6 Department of Educational Development and Research, Faculty of Health, Medicine and Life Sciences, Maastricht University, 6200 Maastricht, Netherlands

**Keywords:** Simulation-based medical education, In situ simulation, Inter-professional, Curriculum, Fidelity, Context, Cross-training

## Abstract

**Background:**

Simulation-based medical education (SBME) has traditionally been conducted as off-site simulation in simulation centres. Some hospital departments also provide off-site simulation using in-house training room(s) set up for simulation away from the clinical setting, and these activities are called in-house training. In-house training facilities can be part of hospital departments and resemble to some extent simulation centres but often have less technical equipment. In situ simulation, introduced over the past decade, mainly comprises of team-based activities and occurs in patient care units with healthcare professionals in their own working environment. Thus, this intentional blend of simulation and real working environments means that in situ simulation brings simulation to the real working environment and provides training where people work. In situ simulation can be either announced or unannounced, the latter also known as a drill. This article presents and discusses the design of SBME and the advantage and disadvantage of the different simulation settings, such as training in simulation-centres, in-house simulations in hospital departments, announced or unannounced in situ simulations.

**Discussion:**

Non-randomised studies argue that in situ simulation is more effective for educational purposes than other types of simulation settings. Conversely, the few comparison studies that exist, either randomised or retrospective, show that choice of setting does not seem to influence individual or team learning. However, hospital department-based simulations, such as in-house simulation and in situ simulation, lead to a gain in organisational learning. To our knowledge no studies have compared announced and unannounced in situ simulation. The literature suggests some improved organisational learning from unannounced in situ simulation; however, unannounced in situ simulation was also found to be challenging to plan and conduct, and more stressful among participants. The importance of setting, context and fidelity are discussed.

**Summary:**

Based on the current limited research we suggest that choice of setting for simulations does not seem to influence individual and team learning. Department-based local simulation, such as simulation in-house and especially in situ simulation, leads to gains in organisational learning. The overall objectives of simulation-based education and factors such as feasibility can help determine choice of simulation setting.

## Background

Simulation-based medical education (SBME) is increasingly recommended, as an educational strategy and for improving patient safety [1–10]. One review concluded that future research should clarify the mechanisms behind effective simulation-based education by asking: “What works, for whom, in what contexts?” [6]. One poorly addressed issue in SBME original research studies and reviews is the choice of context and setting for SBME.

Overall, SBME is a complex educational intervention. SBME was defined by Issenberg et al. as: “In broad, simple terms a simulation is a person, device, or set of conditions which attempts to present education and evaluation problems authentically. The student or trainee is required to respond to the problems as he or she would under natural circumstances” [2]. Simulation techniques and devices can comprise, for example of high-tech virtual reality simulators, full-scale mannequins, plastic models, instructed or standardised patients, animal or animal products, human cadavers, or screen-based simulators. These simulation modalities can be applied in all kinds of simulation settings, and SBME can be applied in various settings target individuals, teams or both, but also aim for organisational learning, such as e.g. practical changes in equipment, guidelines or the physical clinical environment.

Learning in context is a highly discussed topic in medical education [2, 11]. Context can be understood as the circumstances in which a task is undertaken [12]. Various studies indicate that learning can be better applied or recalled when the context and the learning environment resemble the retrieval environment [11, 13, 14]. Medical educators and empirical findings, however, increasingly question this assumption [15–17]. In the following sections we discuss the SBME setting, the design of simulation and the concept of learning in context.

The planning and conduction of SBME may be influenced by the level of fidelity. Fidelity refers to the degree of faithfulness that exists between two entities, and these entities are fundamental for the transfer of SBME and performance in the clinical setting [16]. The notion behind this idea concerning the fidelity of simulation is rooted in the traditional assumption that the closer the learning context resembles the context of practice, the better the learning [14] and is a premise that is discussed below in detail. Fidelity is understood as important in SBME and may improve the effectiveness of a simulation, thereby preparing participants to perform clinically [16]. In the 1990s, the term fidelity was defined in various ways in the flight simulation literature [18], which served as the basis for its later introduction into the medical education literature. The medical educational literature adapted a definition of fidelity divided into two parts [17, 19]: 1) physical or engineering fidelity, which is the degree to which the simulators duplicate the appearance of the real system, and this also covers environmental fidelity; and 2) psychological fidelity, which is the degree to which the simulation participants perceive the simulation as an authentic surrogate for the task being trained. These aspects of fidelity are interrelated, and different modalities of simulation can be combined to increase both physical and psychological fidelity. The complex term, fidelity is discussed in this article with a focus on physical fidelity, i.e. the resemblance of the simulation setting and context to the real setting and context.

### Types of simulation settings

SBME has largely been conducted in an off-site simulation (OSS) setting in simulation centres, which range widely from publically financed simulation centres at hospitals and universities to simulation centres that are detached facilities funded by sponsors and user payment. Some hospital departments also provide OSS as in-house training room(s) specifically set up for simulation training away from the clinical setting but within the hospital department [20–23]. In-house training facilities can be part of hospital departments and resemble to some extent simulation centres but often have fewer technical devices, e.g. permanent audio-visual recording equipment. OSS in-house activities require that departments are able to provide simulation equipment and to ensure that simulation instructors are trained well enough to supply professionally and educationally sound simulations.

Introduced over the past 10 years in situ simulation (ISS) mainly comprises team-based activities that occur in the actual patient care units involving actual healthcare team members in their own working environment [24]. Rosen et al. describe ISS as a blend of simulation and real working environments designed to provide training where people actually work [19]. ISS can also focus on individual skills. ISS can be conducted either announced or unannounced [19, 25], the latter also termed as a drill [25]. Table 1 presents an overview of the different simulation settings.

**Table 1:** Various types of physical simulation settings

Type of physical simulation settings	Description
Off-site simulation in simulation centre	Conducted and placed away from the actual patient care unit; simulation-centres range from publically financed centres at hospitals and universities to simulation centres that are detached facilities funded by sponsors and user payment
Off-site simulation in-house in department	Conducted in training room(s) specifically set up for simulation training away from the clinical setting, but within the hospital in-house training facilities can be part of hospital departments and resemble to some extent simulation centres however often have fewer technical devices, e.g. permanent audio-visual recording equipment
In situ simulation	A blend of simulation and real working environments designed to provide training where people actually work, but ISS can also focus more on individual skills
In situ simulation announced	The staff is informed about the simulation event
In situ simulation unannounced	The involved staff do not have prior knowledge of the event; Can also be termed as a drill

This article discusses the advantages and disadvantages of the choice of simulation setting and the design and delivery of SBME, including choice of target groups, objectives and assessment procedures. We will also provide some tips and share the lessons we have learned, especially when introducing ISS. In this article we focus on postgraduate and interprofessional simulation, and it is beyond focus of the article to discuss simulation for medical or other healthcare professional undergraduate students.

## Discussion

All types of SBME require meticulous planning, which is well described and corroborated by several reviews [2, 3, 8, 9]. To facilitate the discussion about advantages and disadvantages of the choice of simulation setting, Table 2 presents a schematic overview of how simulation settings are potentially related to various components in SBME, which will be discussed in the following. Table 2 is based on various sources and articles, including reviews about ISS [19, 26] and literature specifically addressing randomised and retrospective studies that compare differences in simulation settings [20, 23, 27–29]. The authors went through the literature and discussed and compiled Table 2.

**Table 2:** How various aspects of simulation-based medical education affect the physical simulation setting. Blank spaces indicate that the item has little or no effect; x that the item can have an effect; xx that the item can have a strong effect

	Off-site simulation in simulation centre	Off-site simulation in-house in department	In situ simulation announced	In situ simulation unannounced
1.Less risk of cancellation due to heavy patient load	xx	xx	x	
2.Reported to promote better involvement of all postgraduate healthcare professionals		x	x	x
3.No risk of staff being called away for clinical work	xx	x		
4.Does not require travel time; accessibility for staff easier		xx	xx	xx
5.Popular and promotes recruitment of postgraduate healthcare professionals			x	x
6.Not described as anxiety provoking	x	x	x	
7.May potentially give a greater feeling of safety psychologically	x			
8.Enhances individual learning	x	x	x	x
9.Enhances team learning	x	xx	xx	xx
10.More time potentially set aside, especially for debriefing	xx	x	x	
11.Ideas for organisational changes brought back to the organisation (latent patient safety issues)		x	xx	xx
12.No potential risk of safety hazards due to mixing up medical equipment and utensils	xx	x		
13.No potential risk of unintentional involvement of patients and relatives	xx	xx	x	
14.More efficient use of simulation equipment, which can be shared by many departments, and better facilities to ensure efficient use of high-tech simulation equipment	xx			
15.Potentially more efficient simulations due to development of simulation curriculum	xx	x	x	x
16.Easier access for technicians if simulation equipment has technical problems	xx			
17.Team-based and low-tech simulation can be cheaper due to use of local facilities and equipment		x	xx	xx
18.Potentially more efficient simulations due to better training of simulation instructors	xx	x	x	x

### Choice of learning objectives

Selection the simulation setting for SBME must be guided by the learning objectives. Learning objectives and integration of SBME into the overall curriculum are an essential aspect of curriculum design for every type of educational intervention [30]. As a result, scenarios based on well-defined learning objectives are crucial, and simulation activities can only be as good as the educational programme in which they are embedded [1, 3, 31]. Decades ago, a paper on flight simulation concluded that “The key is the programme, not the hardware” [32], an aspect that Salas et al. also highlight [9]: “Simulators do not make a curriculum, they are merely tools for a curriculum”. Objectives must initially be defined clearly, each of which can focus more on individual or team-based activities, such as communication, cooperation and teamwork, but also on cognitive skills like decision making or on technical and clinical topics. Learning objectives can also be organisational. By organisational learning we mean ideas on organisational and practical changes in e.g. equipment, guidelines and the physical clinical environment [33]. A recent international expert group concluded [10] that system probing, which is an organisational approach, is one of five topics that healthcare simulation can address to improve patient safety. System probing is used to identify patient safety problems that can be improved by training or by system changes and it can serve as a needs assessment and to help define learning objectives and educational interventions [10]. A variety of ISS programmes are designed specifically to test organisational practice [19], i.e. to test new rooms or wards in a hospital [34]. This can, however, cause confusion among participants in a simulation due to the multi-level focus on the individual, team and organisational setup, which is why clearly defined objectives are vital. Learning on an organisational level can differ from individual and team learning [19, 22, 27, 33]. For example, organisational learning can involve changes beyond individual behaviour, like changes in equipment in emergency boxes, in procedures for calling staff and in guidelines [22, 24, 25].

### Target groups

SBME can focus on individual skills training for a specific healthcare professional group or on team training for various healthcare professional groups. Inter-professional simulation is on the agenda in many organisations, which is why it is important to acknowledge that it requires substantial planning and that inter-professional planning requires the use of inter-professional curriculum committees [22, 27, 35]. Boet et al. provide ample information on how to create simulations inter-professionally [35]. Simulations must be developed that provide each healthcare professional group with a significant role to play and involve incorporating a variety of objectives for each group. Adopting this kind of more holistic view is also described as helpful in inter-professional postgraduate simulation [35].

### There is a difference between training and assessment

Be aware of the difference between simulation-based training and simulation-based assessment of simulation participants [30]. In the pre-briefing it is important to tell simulation participants what is expected of them [35]. Assessing participants individually may be relevant and participants who have been tested have been shown to have better retention as a result of what is known as the testing effect [36]. However, some simulation participants may experience that being assessed disrupts the feeling of being in a safe learning environment [37]. Developing a test to be applied in an inter-professional context will, in addition to curriculum development, require the involvement of all the healthcare professional groups that are part of the simulation intervention [38].

Simulation will probably increasingly be used for assessment. In a review Brydges et al. concluded that simulation-based tools may replace work-based assessment of selected procedural skills [7], but McGaghie et al. concluded that less evidence is found on the benefit of SBME in teams as there is still a lack of team-based metrics and standards [4].

With the general move towards more competency-based medical education and workplace-based assessment [39, 40], the role of formative assessment and feedback can be expected to increase. This will likely increasingly blur the line between training and assessment, potentially influencing the role of assessment and the attitudes towards assessment among simulation participants.

### Planning of unannounced in situ simulation (Table 2, point 1)

All simulation requires detailed planning, but particularly unannounced ISS requires multifaceted planning and the need for good management support [22, 26, 29, 41]. Well-established cooperation between educational planners and the departmental management is required and actively involving representatives from all healthcare professional groups results in better planning of postgraduate inter-professional simulation [21, 22, 26–28, 35, 42]. Still, simulation instructors must be prepared to cancel or postpone scheduled unannounced ISS in the event of heavy patient loads or a shortage of staff [22, 43]. The average reported rate of cancellation for unannounced ISS is 28–67% [22, 41, 43] but the percentage seems to go down as training matures [41]. Unannounced ISS must not pose any risk to real-life patients, which means extra staff must replace staff participating in the unannounced ISS [22].

### Healthcare professionals prefeences in simulation (Table 2, point 2–5)

One idea is to make simulation facilities more accessible for all staff in a multiprofessional organisation, which in several articles are an argument for delivering of simulation as ISS and OSS in-house in departments [19–21, 23, 27, 28].

A retrospective study comparing OSS in a simulation-centre with announced ISS found the same outcome in video ratings of team performance in various simulation settings [29]. However, survey-based data showed that participants favoured ISS, which can be seen as an argument to apply ISS to improve recruitment [29].

In a qualitative study staff informed that they had a preconceived preference for participating in ISS because they believed that ISS better matched reality and assumed that this would affect their ability to involve themselves [28].

Some argue in favour of conducting OSS in a simulation centre where the staff cannot be called away for clinical work. The comparison studies on simulation settings [20, 23, 27–29] do not specifically address this issue.

### Some health-care professional experience in situ simulation as anxiety provoking (Table 2, point 6,7)

Some individuals who have participated in unannounced ISS describe it as intimidating [25], but this topic is poorly explored in the literature. One study found that approximately one-third of all staff members thought that unannounced ISS was stressful and unpleasant, despite the fact that all staff members beforehand had been told that a number of unannounced ISS would take place within a specific period [22].

Conducting OSS or an announced ISS can potentially ensure a safer learning environment than unannounced ISS, even though simulation in itself is also reported to be perceived as stressful or intimidating [44]. Boet et al. also reported widespread anxiety concerning inter-professional learning as it entails various difficult interactions involving people from a range of professional groups and perceived status [35]. The precise interplay of the many factors impacting how safe simulation participants feel during simulation remains to be explored. This also underlines the importance of training programmes for simulation instructors [45].

Some argue that potential conflicts of interest from pre-existing personal relationships between simulation instructors and professional healthcare staff can be avoided when simulation is conducted in a simulation centre [46]. This topic is not in focus in any empiric studies.

### Simulation settings do not appear to influence individual and team outcomes (Table 2, point 8–10)

Several non-randomised studies argue that ISS is more effective for learning than OSS because the simulation is conducted in a more authentic environment [24, 41, 47–50]. Little is known about the effect of the physical setting on the practice of simulation [51, 52]. To our knowledge, there are only a handful of studies [20, 23, 27–29] in the medical domain that use randomised or retrospective studies to compare various simulation settings in terms of outcomes. A randomised trial involving training announced ISS versus OSS in-house tested this hypothesis [27]. The ISS and OSS scenarios were identical and standardised, and the simulation instructors were trained to conduct the simulations in a comparative way in both settings. The ISS participants scored the authenticity of the simulation scenarios significantly higher than the OSS participants, but the comparison of ISS versus OSS in-house did not reveal any significant differences regarding all other variables measured, such as individual knowledge, patient safety attitudes, stress measurements, perceptions of the simulations and video-assessed team performance [27]. The findings showed that the only difference was that ISS had an organisational impact. A subsequent qualitative study confirmed that ISS and OSS participants had similar individual and team learning experiences [28].

Another randomised trial comparing OSS in a simulation centre with OSS in-house training showed that the simulation setting was not of importance for the outcome, as expressed by no difference in the acquisition of knowledge and no differences in completion for basic tasks and teamwork [20, 23].

Some argue that more time is potentially set aside, especially for debriefing in OSS [46]. However this is not addressed in empiric studies. The time-issue in unannounced ISS is clear [22, 41, 43], and less time is maybe therefore spent on debriefing.

To our knowledge there are no studies comparing announced and unannounced ISS. Further studies are also needed that include outcome on long-term retention and patient-based outcomes.

### In situ simulation achieves greater learning at the organisational level (Table 2, point 11)

Simulation can be used to test equipment, new procedures and physical environments. Articles on ISS discuss the value of ISS for identifying latent safety threats in organisations [19, 24, 27, 41, 47, 53]. Testing equipment and procedures can take place in simulation centres, but the literature focuses on ISS. Studies describe how ISS can successfully be used to test the renovation of wards and the construction of new wards [34, 54–57] or to determine how to perform individual procedures [56].

A randomised trial and a subsequent qualitative study confirm that more information on organisational deficiencies comes from ISS participants compared to OSS participants in-house [27, 28]. OSS in-house training is described as useful for identifying organisational deficiencies [21, 27, 28, 58], but the ISS setting in particular provides more information than OSS on deficiencies concerning technology and tools [27, 33].

### In situ simulation and in-house simulation may compromise patient safety (Table 2, point 12-13)

A potential disadvantage of doing simulations that take place outside a simulation centre is that ISS and OSS in-house can compromise patient safety [59]. For example medication prepared for ISS or OSS in-house can potentially get mixed up with real medication, or equipment used for ISS might be returned without being made ready for use in real clinical situations [46, 59]. Using labels marked “Simulation only” can be a precaution that can be taken to avoid these problems. ISS will most often involve the use of equipment from the clinical site, thus making it simpler to plan, whereas OSS in-house simulation instructors must organise all relevant equipment. Conducting OSS in-house and ISS requires storage space for equipment, and simulation instructors have to schedule time to organise mannequins and equipment. Faculty planning simulations must also incorporate clean-up procedures and an awareness among simulation instructors of how patient safety can be compromised due to poor planning [59]. ISS can also potentially upset patients [59], but providing useful information for patients and relatives may also result in a positive effect. Signage can help them to recognise the training nature of the activities. If a research approach is taken in this new process, knowledge on the perspective of patients and relatives can be gathered.

### Simulation can be used to test facilities in new building facilities

A more recently applied use of OSS modalities can involve using a mock-up or sandbox technique [60, 61] when constructing and testing new facilities. The mock-up technique is a 1:1 construction of a unit or other rooms that allows architects and designers, in cooperation with clinical staff, to test ideas and solutions [60]. The sandbox technique allows staff to practice new care delivery in new buildings [61]. It is important to apply these simulation methods in the early phases of planning and decision making when building new wards and hospitals. Recent literature on the design of new hospitals stresses the lack of integration between physical learning spaces and underlying teaching strategies [62].

### Integration of simulation into the educational strategy of departments

Simulation is expected in the future to be an increasingly recommended educational strategy for all healthcare professionals, just as an increase in inter-professional simulation programmes is expected [35]. Practicing teamwork integrated with simulation-based skills training that encompasses a clinical approach is preferable and has been shown to be associated with significant improvements [37, 58, 63, 64].

One idea is to make simulation facilities more accessible for staff and to integrate simulation into the educational strategy of departments. This approach can prevent simulation sessions from becoming stand-alone events [35], and establishing simulation rooms when constructing new hospitals should be considered. These rooms should preferably be located close to departments where various specialties work together and team training can take place. New wards, emergency rooms, operating theatres and delivery wards can also be designed to facilitate ISS, e.g. in the form of video-recording equipment and rooms nearby for debriefing.

### Sharing facilities and simulation equipment (Table 2, point 14–17)

Cooperation between departments can enable better use of rooms and simulation equipment. Further coordination between local simulation in hospital departments and simulation centres will help to avoid the purchase of equipment that will be underutilised and contribute to relevant access to technicians. Department-based simulations could be supported by simulation centres to ensure that simulation programmes are adequately developed and standardised. Further this might help to guarantee that simulation instructors are sufficiently trained, in addition to encouraging and coordinating simulation research [45, 46]. Research on inter-professional postgraduate simulation shows that simulation conducted in close proximity to the clinical setting has a positive impact and that the departments involved gain useful organisational information for improving care [20, 21, 23, 27, 28, 37, 58, 63, 64], which are arguments for incorporating simulation facilities in new hospitals.

Simulation-based activities involving high-tech simulation for technically advanced clinical procedures are most often centralised in simulation centres due to the advanced level of the simulators and the requirements they pose on their users [65].

Although several studies show that successful ISS can take place with at a minimal cost compared to simulation centres [19, 29, 66–68], ISS can require extra space for clinical activities, which may mean increased costs.

### Discussion of setting, context and physical fidelity

To some extent, this article uses the term setting synonymously with context or physical surroundings. However, context can be expanded to also include more than the physical context, i.e. the semantic and commitment context [15]. Semantic context reflects how well the context contributes to the learning task while commitment context reflects motivation and responsibility [15]. One argument in favour of ISS is the contextual similarity to the context of working. The rooms and the equipment, for example are “real”, even though they are used for simulation purposes [19, 47, 69].

Many argue for learning in context [2, 11] based on various studies [11, 13, 14]. Some medical educators question whether fidelity plays a prominent role in the context [15–17]. The notion behind the idea of fidelity is that the more closely the simulation resembles the context of practice, the better the learning. However, the comparison studies on settings for simulation described in this article [20, 23, 27–29] indicate that the physical context or physical fidelity of the simulation setting, such as OSS or ISS, is not the most important aspect for individual and team learning, indicating that the semantic and motivational context can be more important.

### Perspectives on future research

In general, we found that choice of setting does not seem to influence individual and team learning; however, future research would benefit from collaboration between medical education researchers and practical organisers of simulations as more research is necessary to better understand what additional aspects of simulation are fundamental for learning.

Carrying out simulation is costly and SBME is also expected to increase substantially in the coming years. Specific areas that would benefit from future research include the implementation of simulation [70] and the interplay between and the role of local organisers of simulations and of simulation centres. Research would profit greatly by encouraging collaboration between practical organisers of simulations and medical education researchers. Based on our studies the use of cross training was ill-advised [27, 28], but more research is warranted that involves groups beyond the postgraduate multi-professional teams we examined.

The term sociological fidelity has recently been introduced in the field of simulation and expresses the interactions between learners in order to create authenticity with high levels of social realism [35, 42]. Additional research on sociological fidelity may be relevant as factors related to the interaction between simulation participants appear to be of more importance than the simulation’s physical setting. The current understanding of fidelity as physical and psychological fidelity is under debate [16, 17, 52, 71] and may not be adequate enough to explain the learning-relevant processes in inter-professional simulation. Discussing the importance of social practice, hierarchy, power relations and other factors affecting inter-professional teamwork is rather new in the simulation literature [35, 42, 52, 72] and exploring concepts like sociological fidelity may prove useful in future research on simulation.

Situativity theory [13] argues that knowledge, thinking and learning are situated in experience [11, 13, 73]. However, results from the above-mentioned comparison studies [20, 23, 27–29] on different simulation settings seem to show that some of the physical aspects of the simulation setting play a minor role compared to other factors. This assumption appears to be partly inconsistent with situated learning theory, which states that increased fidelity leads to improved learning [13], but does not consistently appear to be the case for physical fidelity. Future research could help to more sharply define what influences the learning context.

Participants in postgraduate simulation thought that participating in authentic teams in their own roles as healthcare professionals was important [27, 28]; however, we need to know if this perception affects learning and clinical performance. This perception stands in contrast to the premise behind cross training, which is recommended in the simulation literature [3, 74]. Cross training is defined as “an instructional strategy in which each team member is trained in the duties of his or her teammates” [75]. It is argued that if all team members have a shared understanding of other people’s roles, the risk of making errors decreases. Although there are empirical studies that address cross training, they only comprise small teams in an experimental laboratory setting and, to our knowledge, no medical studies have been undertaken that involve postgraduate multi-professional medical teams [74–76].

## Conclusions

Based on the current limited research [20, 23, 27–29], we conclude that the choice of physical setting for simulations does not seem to influence individual and team learning. Department-based local simulation, such as OSS in-house and especially ISS, leads to gains in organisational learning, and unannounced ISS appears to provide more organisational learning than announced ISS [27, 28]. The overall objectives and aim of a simulation and factors such as feasibility can help determine which simulation setting to choose. Studies on postgraduate inter-professional training show that local training, such as announced and unannounced ISS or OSS in-house, offers various advantages, e.g. locally run courses benefit local organisational learning, reduce costs and increase the accessibility of training for professional staff [37, 58, 63, 64]. Some of the potential disadvantages of holding courses locally can be organisational problems and poor quality content due to badly organised simulations and a lack of qualified simulation instructors. These disadvantages need to be specifically addressed, and explicit collaboration and coordination between the organisers of local simulation and simulation centres can be recommended and may help avoid some of these issues. The advantages and disadvantages of announced and unannounced ISS are poorly explored in the literature, but some individuals who have participated in unannounced ISS describe it as intimidating, and unpleasant [22, 25].

## Abbreviation

ISS: In situ simulation
OSS: Off-site simulation
SBME: Simulation-based medical education
